# Ultrastructure of Calcareous Dinophytes (*Thoracosphaeraceae*, *Peridiniales*) with a Focus on Vacuolar Crystal-Like Particles

**DOI:** 10.1371/journal.pone.0054038

**Published:** 2013-01-08

**Authors:** Carmen Zinssmeister, Helmut Keupp, Gilbert Tischendorf, Freya Kaulbars, Marc Gottschling

**Affiliations:** 1 Department Biologie, Systematische Botanik und Mykologie, Ludwig-Maximilians-Universität München, München, Germany; 2 Freie Universität Berlin, Fachbereich Geologische Wissenschaften, Fachrichtung Paläontologie, Berlin, Germany; 3 Freie Universität Berlin, Fachbereich Biologie, Chemie, Pharmazie, Institut für Biologie – Mikrobiologie, Berlin, Germany; RMIT University, Australia

## Abstract

Biomineralization in calcareous dinophytes (Thoracosphaeracaea, Peridiniales) takes place in coccoid cells and is presently poorly understood. Vacuolar crystal-like particles as well as collection sites within the prospective calcareous shell may play a crucial role during this process at the ultrastructural level. Using transmission electron microscopy, we investigated the ultrastructure of coccoid cells at an early developmental stage in fourteen calcareous dinophyte strains (corresponding to at least ten species of *Calciodinellum*, *Calcigonellum*, *Leonella*, *Pernambugia*, *Scrippsiella*, and *Thoracosphaera*). The shell of the coccoid cells consisted either of one (*Leonella*, *Thoracosphaera*) or two matrices (*Scrippsiella* and its relatives) of unknown element composition, whereas calcite is deposited in the only or the outer layer, respectively. We observed crystal-like particles in cytoplasmic vacuoles in cells of nine of the strains investigated and assume that they are widespread among calcareous dinophytes. However, similar structures are also found outside the Thoracosphaeraceae, and we postulate an evolutionarily old physiological pathway (possibly involved in detoxification) that later was specialized for calcification. We aim to contribute to a deeper knowledge of the biomineralization process in calcareous dinophytes.

## Introduction

Biomineralization is defined as the fundamental biological process by which living organisms produce minerals, often to harden or stiffen existing tissues or subcellular organic matrices. Mineralized structures have evolved multiple times independently and are taxonomically widely distributed over the tree of life. Subsequently, many similar cellular steps take place in distantly related lineages [1]–[4], and the resulting, occasionally complex crystal architectures may have multifunctional properties. The biomineralization process and its structural basis are well understood in metazoans including mollusks, corals, and vertebrates [5]–[7], but also in such protists as foraminifers and coccolithophores [8]–[10]. Mineralized structures are likewise found in some taxa of the unicellular Dinophyceae (Alveolata), where the mechanisms of crystal formation are largely elusive.

Many dinophytes develop at least two principally different stages during their life history: a phototrophic, motile cell (i.e., the theca, composed of cellulose plates that are formed in amphiesmal vesicles) and an immotile coccoid cell (commonly termed a ‘cyst’). In the calcareous dinophytes (Thoracosphaeraceae, Peridiniales), it is particularly the shell of the coccoid cells that is mineralized by calcitic crystals [11]–[12]. Shell morphology and ultrastructure is diverse among calcareous dinophytes [13], and many species have been described, particularly from the fossil record [14]–[16]. As the potential to form calcareous structures is unique within the entire alveolates, it has been considered an apomorphic character trait supporting the monophyly of the Thoracosphaeraceae [17]. This assumption has gained some corroboration from molecular sequence data, although a number of (presumably secondarily) non-calcareous taxa might be also included in this group [18]–[20]. Molecular phylogenies segregate calcareous dinophytes into three main lineages, namely the E/Pe-clade (for *Ensiculifera* Balech, 1967 and *Pentapharsodinium* Indel. & A.R.Loebl.), the T/Pf-clade (for *Thoracosphaera* Kamptner and *Pfiesteria* Steid. & J.M.Burkh.), and *Scrippsiella* Balech ex A.R.Loebl. *sensu lato* (*s.l*.).

Since the early studies of dinophyte anatomy using transmission electron microscopy (TEM) [21]–[23], much progress has been made in understanding their complex and diverse organizations at the subcellular level. Much attention has been given to the ‘apical furrow’ system [24]–[25] and the flagellar apparatus [26]–[29], but the biomineralization process in calcareous dinophytes has not been the focus of such studies. Ultrastructure investigations into the subcellular components involved in biological processes such as the encystment of cells expand the basic data necessary for robust phylogenetic reconstructions.

Immature coccoid cells of *Scrippsiella minima* X.Gao & J.D.Dodge, in which the initial phase of mineralization takes place, are surrounded by two continuous matrices of unknown compounds that are delineated by an outer, middle, and inner unit membrane [30]. It has been assumed that calcification proceeds within the outer matrix, starting at protrusions composed of fibrous material. In coccoid cells of *Thoracosphaera heimii* (Lohmann) Kamptner, a single matrix develops, delineated by an outer and inner unit membrane [31]–[32]. The matrix is filled with numerous large, regularly arranged crystals in mature coccoid cells, while small, cylindrical seed crystals are found in immature coccoid cells. Inouye and Pienaar [31] have studied the crystallization process in *Th. heimii* in more detail and have discovered small and large cytoplasmic vesicles (or vacuoles) likewise containing cylindrical crystals. Such vacuoles may derive from the Golgi apparatus and actively transport seed crystals from the center towards the periphery of the cell. ‘Crystal-like bodies’ similar in appearance have been reported outside the calcareous dinophytes, including other Peridiniales [33]–[35] and Suessiales [24], [36].

In this study, we investigate the ultrastructure of several calcareous dinophytes to document the subcellular structures that may play a role in biomineralization. Elbrächter and colleagues [37] have identified this field as one of the most serious gaps in knowledge in their Agenda Calcareous Dinophytes. We focus on immature coccoid cells because they are the likely stages, in which such structures can be observed. The study of (even immature) coccoid cells is challenging, as fixatives frequently do not penetrate the shell [30], [38]. Nevertheless, we have found vacuoles containing crystal-like particles comparable to *Thoracosphaera* [31] also in other species. We aim to contribute to a more complete understanding of the biomineralization process in calcareous dinophytes.

## Materials and Methods

### Morphology

Fourteen calcareous dinophyte strains were collected and isolated from environmental samples (Table 1). They were cultivated in a climate chamber Percival I-36VL (CLF PlantClimatics; Emersacker, Germany) at 18°C or 23°C, 80 μmol photons m^−2^ s^−1^, and a 12∶12 h light: dark photoperiod by using K-Medium without silicate [39] and 35 or 30 psu artificial seawater (hw marinemix professional: Wiegandt; Krefeld, Germany) at pH 8.2. Strains are currently held in the culture collections at the Institute of Historical Geology/Palaeontology (University of Bremen, Germany) and the Institute of Systematic Botany and Mycology (University of Munich) and are available upon request.

**Table 1:** Species list of TEM investigations (abbreviations: n.i., not indicated).

Strain No.	Taxonomy	Species name with author	Locality	Lat.	Long.	Collector
GeoB 110	*Scrippsiella*	*Calcigonellum infula* Deflandre, 1949	Mediterranean Sea (Spain)	41°21′N	3°01′E	n.i.
SZN#74	*Scrippsiella*	*Calciodinellum operosum* Deflandre, 1947	Mediterranean Sea (Italy)	40°43′N	14°10′E	Montresor
GeoB 34	*Scrippsiella*	*Calciodinellum* aff. *Operosum* Deflandre, 1947	Middle Atlantic	08°30′N	32°27′W	n.i.
tub*2	*Scrippsiella*	“*Calciodinellum*” spec.	Eastern South Pacific (Chile)	28°15′S	78°00′W	n.i.
GeoB 38	T/Pf	*Leonella granifera* (D.Fütterer) Janofske & Karwath	Western Atlantic (Brazil)	06°57′N	47°54′W	n.i.
GeoB*61	*Scrippsiella*	*Pernambugia tuberosa* (Kamptner) Janofske & Karwath	South Atlantic (Brazil)	11°32′S	28°35′W	n.i.
GeoB 411	*Scrippsiella*	*Scrippsiella bicarinata* Zinssmeister, S.Soehner, S.Meier & Gottschling	Mediterranean Sea (Italy)	41°15′N	13°36′E	Gottschling, Zinßmeister & Söhner
GeoB*185	*Scrippsiella*	*Scrippsiella trochoidea* (F.Stein) A.R.Loebl.	Baltic Sea (Germany)	54°22′N	10°09′E	Meier
GeoB 188	*Scrippsiella*	*Scrippsiella* aff. *Trochoidea* (F.Stein) A.R.Loebl.	Mediterranean Sea (France)	42°28′53′′N	3°08′E	Gottschling
GeoB 283	*Scrippsiella*	*Scrippsiella* aff. *trochoidea* (F.Stein) A.R.Loebl.	North Sea (Norway)	63°28′N	9°25′E	Gottschling & Petersen
GeoB 377	*Scrippsiella*	*Scrippsiella* aff. *trochoidea* (F.Stein) A.R.Loebl.	Mediterranean Sea (Italy)	40°40′N	14°46′E	Gottschling, Zinßmeister & Söhner
M34*25/5	*Scrippsiella*	*Scrippsiella* aff. *trochoidea* (F.Stein) A.R.Loebl.	South Atlantic (Guyana)	11°54′N	57°48′90′′W	n.i.
GeoB 228	*Scrippsiella*	*Scrippsiella trochoidea* var. *aciculifera* Montresor	Mediterranean Sea (Italy)	40°07′N	17°19′E	n.i.
GeoB 211	T/Pf	*Thoracosphaera heimii* (Lohmann) Kamptner	Eastern Mediterranean Sea	–	–	n.i.

Cultivated living cells were observed using an Olympus CKX41 inverse microscope, equipped with a Kappa camera DX 20H-FW (supplied with Calypso software). For scanning electron microscopy (SEM) preparation, coccoid cells were desalinated in bi-distillate water and air-dried on a glass slide that was fixed on a SEM stub (details are given in [40]). Samples were sputter-coated with platinum and documented with a LEO 438 VP (Zeiss) SEM.

### Transmission electron microscopy (TEM)

For TEM standard protocols, large thecate and coccoid cells (with a focus on early stages of the encystment) were fixed with 2.5% glutaraldehyde (Plano; Wetzler, Germany) in 0.2 M cacodylate buffer (Roth; Karlsruhe, Germany) and 0.25 M saccharose (Roth) at pH 8.0 for 1.5 h. An alternative protocol fixed the cells directly in cultivation media that were afterwards transferred to 0.2 M cacodylate buffer and 0.25 M saccharose at pH 8.0. After fixation, the cells were washed in 0.2 M CaCO_3_ buffer at pH .0 in a graded saccharose series (0.125 M, 0.05 M, 0.025 M, 0.01 M, without) each for 15 min and post-fixed with 1% osmiumtetroxide (Science Services; Munich, Germany) in 0.2 M cacodylate buffer. Following the instructions of the Embedding Medi Kit (Science Services), samples were dehydrated in a graded ethanol (Roth) or acetone (Roth) series (30%, 50%, 70%, 90%, 100%, 100%, 100%), and gradually infiltrated and embedded in Spurr's resin [41].

The largest thecae and cells with a roundish form even as large as coccoid stages were selected for sectioning. Ultrathin sections were prepared with an ultra microtome (Leica EM UC6 Ultramikrotom). Sections were spread with 99% chloroform (Roth) and collected on copper 200 square mesh and 200 single bar grids (Plano) covered with collodium. Grids were stained with 1% aqueous uranylacetate (Plano) for 2 min and lead citrate (Plano) for 4 min [42]. TEM observations were done using a FEI Morgagni or a Zeiss EM 912.

## Results

### Cell morphology and life history

*Leonella granifera* (Fütterer) Janofske & Karwath and *Th. heimii* (both members of the T/Pf clade) produced mainly coccoid cells dividing vegetatively (thecate cells were rarely found under cultivation conditions). All investigated species of *Scrippsiella s.l*. (i.e., including also those of *Calcigonellum* Deflandre, 1949, *Calciodinellum* Deflandre, 1947, and *Pernambugia* Janofske & Karwath) developed golden brown, photosynthetically active thecate cells, which were always abundant under cultivation conditions because of vegetative division. The shape of the thecate cells was spherical to ovoid, with a rounded to conical apex. Early coccoid cells developed after shed of the theca by ecdysis and were darker than the thecate cells from the beginning. A red accumulation body was often already visible at this stage. Coccoid cells appeared first hyaline and darkened towards the brownish-opaque color at maturity after a few minutes.

**Figure 1:**
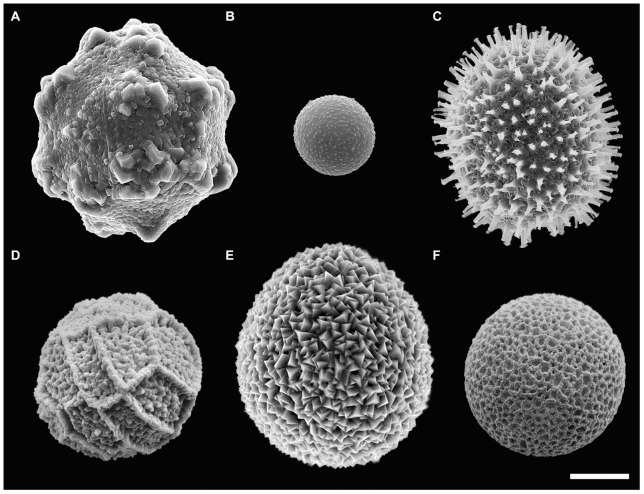
Morphological diversity of calcareous coccoid cells. (SEM). A: *Scrippsiella bicarinata* (GeoB 411, note the bicarinate tabulation resulting from the fusion of pre- and postcingular plate equivalents). B: *Thoracosphaera heimii* (CCCM 670, note the small size). C: *Scrippsiella trochoidea* (GeoB*185, note the spiny surface). D: *Calciodinellum operosum* (SZN#74, note the holotabulate tabulation). E: *Scrippsiella* aff. *trochoidea* (GeoB 283, note the spiny surface). F: *Calciodinellum* aff. *operosum* (GeoB 34, note the smooth surface). Scale bar: 10 µm.

Figure 1 shows a SEM image selection of the calcareous coccoid cell diversity investigated here with respect to their ultrastructure. Mature coccoid cells varied in size across species and were spherical to ovoid. The shell surface likewise differed between taxa, ranging from smooth without ornamental structures (*Th. heimii*: Fig. 1B) to reticulate (*Calciodinellum* aff. *operosum* Deflandre, 1947: Fig. 1F), spiny [*Scrippsiella trochoidea* (F.Stein) A.R.Loebl.: Fig. 1C,E9, imperfectly intratabulate (*Scrippsiella bicarinata* Zinssmeister, S.Soehner, S.Meier & Gottschling: Fig. 1A) to holotabulate (*C. operosum*: Fig. 1D). In some cases, remnants of an outer membrane covering the coccoid cell were present, and this membrane was always entirely intact in *Th. heimii* (Fig. 1B) and *L. granifera*.

### Comparative ultrastructure

**Figure 2:**
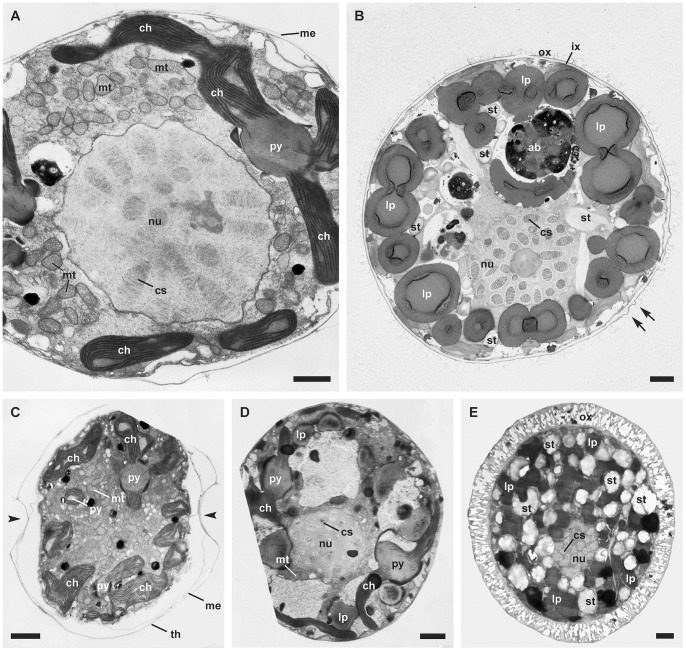
General ultrastructure. (TEM). A. Large thecate cell of *Scrippsiella bicarinata* (GeoB 411, note the peripheral chloroplast connected to a multiply-stalked pyrenoid). B. Early coccoid cell of *Pernambugia tuberosa* (GeoB*61, note the numerous peripheral lipid droplets, the two matrices surrounding the cell and the protrusions highlighted by arrows). C. Large thecate cell (longisection) of *Scrippsiella bicarinata* (GeoB 411, note the multiply-stalked pyrenoids and the numerous mitochondria in the center of the cell). D. Early coccoid cell of *Scrippsiella* aff. *trochoidea* (GeoB 283, note the chloroplasts with stalked pyrenoids and the lipid droplets). E. Mature coccoid cell of *Leonella granifera* (GeoB 38, note the numerous starch grains and lipid droplets and that the cell is surrounded by a single layer containing large, regularly arranged calcareous crystals). Abbreviations: ab, accumulation body; ch, chloroplast; cs, chromosomes; ix, inner matrix; lp, lipid droplet; nu, nucleus; mt, mitochondrion; ox, outer matrix; py, pyrenoid; st, starch grain. Scale bars: 2 µm.

**Figure 3:**
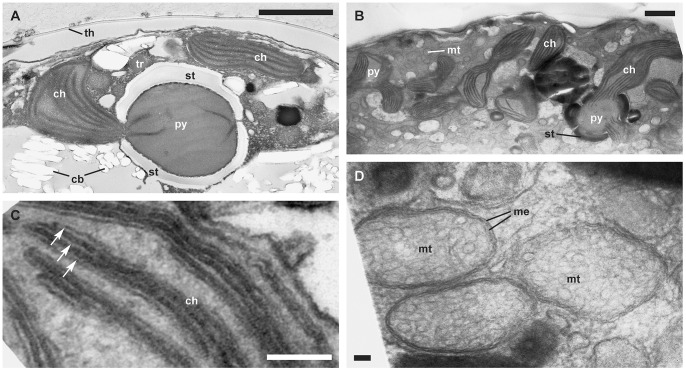
Ultrastructural traits in detail. (TEM). A. Multiply-stalked pyrenoid covered by a starch shed of *Calciodinellum* aff. *operosum* (GeoB 34, note the large, vacuolar crystal-like particles). B. Different chloroplast types of *Scrippsiella bicarinata* (GeoB 411, note that chloroplasts could be connected to multiply-stalked pyrenoids covered by a starch shed, or have interlamellar pyrenoids, with thylakoid lamellae leading through the pyrenoid. C. Two to four thylakoid lamellae (arrows) of *Scrippsiella bicarinata* (GeoB 411). D. Mitochondria with tubular cristae of *Scrippsiella* aff. *trochoidea* (GeoB 283). Abbreviations: ch, chloroplast; cb, crystal-like particle; me, membrane; mt, mitochondrion; py, pyrenoid; st, starch grain; tr, trichocyst. Scale bars: A and B 1 µm, C and D 0.1 µm.

**Figure 4:**
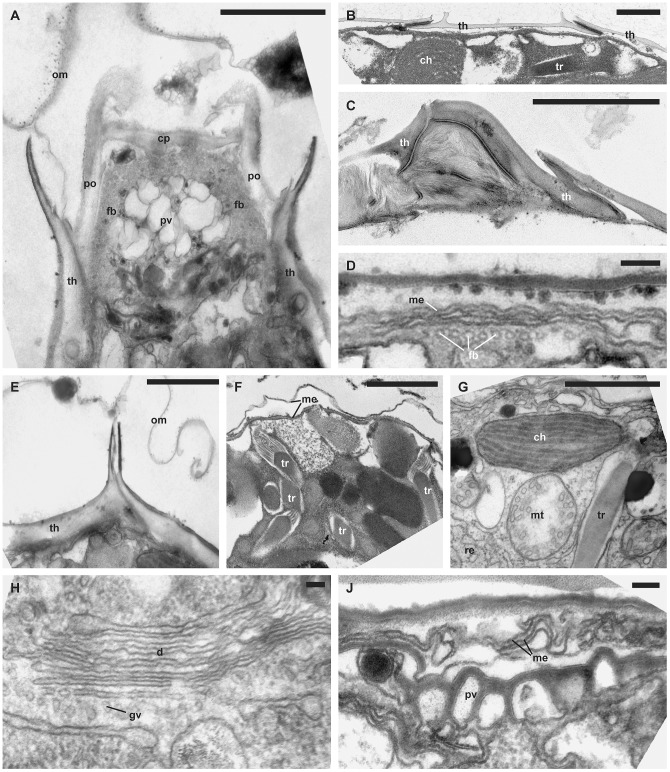
Ultrastructural traits in detail. (TEM). A. ‘Apical furrow’ system of *Scripsiella trochoidea* (GeoB 377, note the numerous vesicles under the cell surface). B. Overlapping thecal plates of *Scripsiella trochoidea* (GeoB 188, note the outer protrusions of overlapping theca plates). C. Overlapping theca plates in the sulcal region of “*Calciodinellum*” spec. (tub*2). D. Strand of peripheral microtubules in *Scrippsiella bicarinata* (GeoB 411, note the multiple membranes under the cell surface). E. Thecal plate boundary of *Scripsiella trochoidea* (GeoB 377, note the detached outer unit membrane). F. Trichocysts of *Scrippsiella trochoidea* var. *aciculifera* (GeoB 228). G. Subcellular organization of *Scrippsiella bicarinata* (GeoB 411, note the longisection of a trichocyst and the rough endoplasmatic reticulum indicated by an arrow). H. Golgi apparatus of *Scrippsiella trochoidea* (M34*25/5). J. Pusule of *Scrippsiella bicarinata* (GeoB 411, note the multiple membranes under the cell surface). Abbreviations: cb, crystal-like particle; ch, chloroplast; cp, cover plate; d, dictyosome, fb, microtubular fiber; gv, Golgi-derived vesicle; me, unit membrane; mt, mitochondrion; po, pore plate; pv, pusular vesicle; py, pyrenoid; re, rough endoplasmatic reticulum; st, starch grain; tr, trichocyst; th, thecal plate. Scale bars: A to C and E to G 1 µm, D, H, J 0.1 µm.

Cell ultrastructure was largely similar in organization among different calcareous dinophyte species (Figs. 2–4). Thecate cells were always smaller than coccoid cells within particular strains. Thecal plates were surrounded by a unit membrane (Figs. 2A, 4A,E), which was visible particularly at their boundaries (Figs. 2C, 4E). Chloroplasts, mitochondria, and other compartments such as trichocysts were likewise present in all of the cells examined.

**Figure 5:**
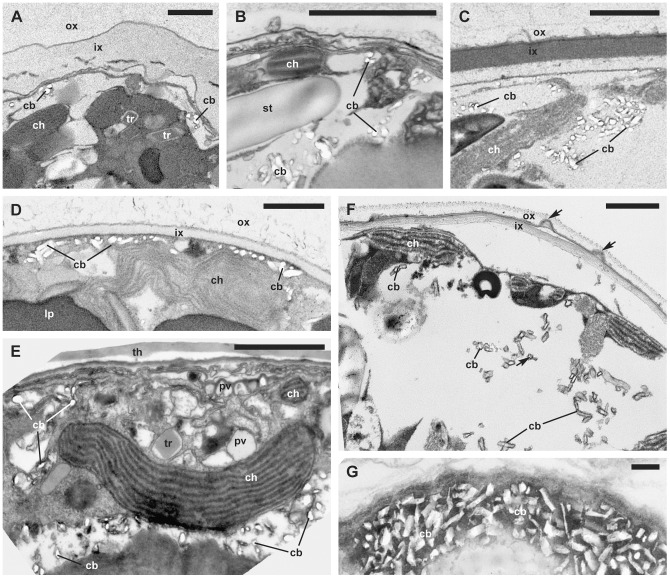
Vacuolar crystal-like particles. (TEM). A. *Scrippsiella trochoidea* var. *aciculifera* (GeoB 228, note the two matrices surrounding the cell, the middle unit membrane is partly disbanded). B. *Thoracosphaera heimii* (GeoB 211, note the single matrix that will calcify). C. *Calcigonellum infula* (GeoB*110, note the two matrices surrounding the cell). D. *Pernambugia tuberosa* (GeoB*61, note the two matrices surrounding the cell). E. *Scrippsiella bicarinata* (GeoB 411, early coccoid cell, the theca is still attached). F. *Calciodinellum* aff. *operosum* (GeoB 34, note the two matrices surrounding the cell and the protrusions between the outer and inner matrix indicated by arrows). G. *Calciodinellum operosum* (SZN#74, note the large crystal-like particles). Abbreviations: cb, crystal-like particle; ch, chloroplast; ix, inner matrix; ox, outer matrix; pv, pusular vesicle; py, pyrenoid; st, starch grain; tr, trichocyst; th, thecal plate. Scale bars: 1 µm.

In all of the thecate cells investigated (Fig. 2), many large chloroplasts were present in peripheral positions. Moreover, different types of pyrenoids were found within those cells that showed structural associations with the chloroplasts. Some large chloroplasts constituted a network, as they were connected by multiply-stalked pyrenoids (Figs. 2A,C–D, 3A–B). Additionally, starch grains adjacent to pyrenoids were present (Fig. 3A). Smaller chloroplasts showed internally fusiform, interlamellar pyrenoids (Figs. 2A,C, 3B). Some chloroplasts were neither attached to pyrenoids nor to starch grains and were particularly small in size. (Figs. 3A, 5E). The thylakoid lamellae were more or less parallel to each other, an arrangement that was occasionally perturbed by the presence of pyrenoids (in which case the lamella fibers led into the pyrenoids). The thylakoids consisted of two to four lamellae (Figs. 2A, 3C, 4G).

Oval to elongated mitochondria with tubular cristae and surrounded by two unit membranes were visible (Fig. 3D). They were numerous and distributed all over the cytoplasm of thecate cells (Fig. 2A,C). The globose dinokaryon surrounded by two unit membranes was located close to the center of the cell and always showed condensed, rod-shaped chromosomes (Figs. 2A–B,D,E). The Golgi apparatus was located in the center of the cell close to the dinokaryon and consisted of a stack of few, flattened cisterns of the dictyosome. Golgi-derived vesicles were likewise visible near the dictyosome (Fig. 4G). A roundish accumulation body (Fig. 2B) was often present in large thecate cells and was always developed in coccoid cells.

**Figure 6:**
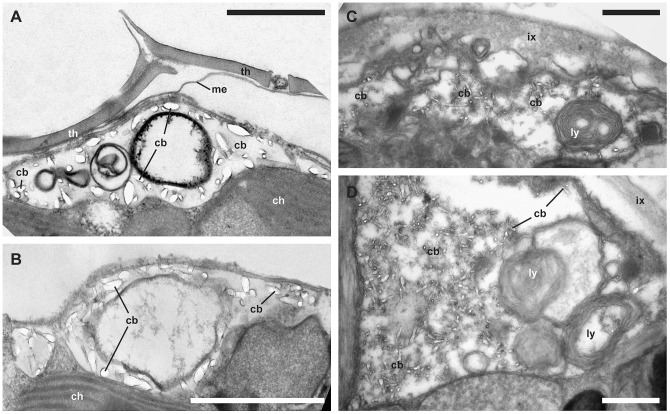
Vacuolar crystal-like particles. (TEM). A: *Scrippsiella trochoidea* (GeoB*185, early coccoid cell, the theca is still attached; note the intravacuolar vesicles). B. *Scrippsiella trochoidea* (GeoB*185). C. *Scrippsiella* aff. *trochoidea* (GeoB 283, note the lysozymes as intravacuolar membrane whorls). D. *Scrippsiella* aff. *trochoidea* (GeoB 283, note the lysozymes as intravacuolar membrane whorls). Abbreviations: cb, crystal-like particle; ch, chloroplast; ix, inner matrix; ly, lysosme; me, unit membrane; th, thecal plate. Scale bars: 1 µm.

In *S*. aff. *trochoidea*, a remarkably high number of lysozymes (Fig. 6C,D) were scattered throughout the cytoplasm and were even found within vesicles. The pusular system was formed by tubules of about 50 to 100 nm (Figs. 4A,H), and vesicles leading close to the flagellar base. Under the plasmalemma, cellulose plates were present that overlapped at their boundaries (Figs. 4A–E). The apical furrow system consisted of the apical pore plates, and the pore itself was covered by one such plate (Fig. 4A). The sulcal region consisted of few overlapping sulcal plates (Fig. 4C). Under the thecal plates, a few unit membranes were sometimes visible (Figs. 4, 6C,D). Microtubular fibers were located near the cell periphery, either as part of the cytoskeleton (Fig. 4A) or functioning as anchorage for the flagellar apparatus (Fig. 4D).

Before encystment and particularly in cells of the coccoid stage, size and number of chloroplasts decreased. Moreover, the number of starch grains and lipid droplets (both with a storage function) increased during encystment (Figs 2B,D) and in coccoid cells (Fig. 2E). At early stages of encystment, the species of the *Scrippsiella s.l*. lineage exhibited two layers, an outer and an inner organic matrix (Figs. 2B, 5A,C–D,F, 6C,D) of unknown composition. Between these two layers, small protrusions could occasionally be detected (Figs. 2B, 5F). By contrast, early coccoid cells of *L. granifera* (Fig. 2E) and *Th. heimii* (both belonging to the T/Pf-clade) were surrounded by a single organic matrix. Directly under this layer, protrusions similar in appearance to those found in *Scrippsiella s.l*. were present (Fig. 2B). Mature coccoid cells were usually not suitable for TEM, except those of *L. granifera* (Fig. 2E). The calcitic crystals deposited in the single matrix surrounding the cell were not preserved after the treatment with uranylacetate and lead citrate and were therefore recognized as empty space in the thin sections.

### Crystal-like particles and biomineralization

In nine of fourteen strains (corresponding to at least eight species), crystal-like particles were detected in encysting cells. They were found within cytoplasmic vacuoles, which were peripherally located in the cell body. Such vacuoles were variable in size and were particularly large in *Calcigonellum infula* Deflandre, 1949 (Fig. 5C), *C*. aff. *operosum* (Fig. 5F), and *S*. aff. *trochoidea* (Fig. 6C–D). The crystal-like particles in the vacuoles were between 50 to 380 nm in length and 24 to 86 nm in width. They had an elongated and cylindrical shape and were irregularly scattered with sometimes large gaps between each other. Only *C. operosum* (Fig. 5G) showed large, densely arranged crystal-like particles of varying size (330 to 1200 nm long and 70 to 470 nm wide) and shape, ranging from rod-like to cylindrical to square-cut. Crystal-like particles of *Pernambugia tuberosa* (Kamptner) Janofske & Karwath (Fig. 5D), *S. bicarinata* (Fig. 5E), *S. trochoidea* (Fig. 6A–B), *S. trochoidea* var. *aciculifera* Montresor (Fig. 5A), and *Th. heimii* (Fig. 5B) had an ovoid to barrel-like shape with blunt edges. In *C. infula* (Fig. 5C), *C*. aff. *operosum* (Fig. 5F), and *S*. aff. *trochoidea* (Fig. 6C–D), the shape of the crystal-like particles was square-cut.

The crystal-like particles showed some structural association to the vacuolar membrane (in, e.g., *P. tuberosa*: Fig. 5D and *S* *. trochoidea*: Fig. 6A–B) and/or internal membranes of the vacuoles (in, e.g., *S. bicarinata*: Fig. 5E and *Th. heimii*: Fig. 5B). In *S*. aff. *trochoidea* (Fig. 6C–D) and *C. operosum* (Fig. 5G), the crystals seemed to be present in higher number and surrounded by dense material of unknown origin, possibly unit membranes. A connection between membranes and crystal-like particles was not detected in *C. infula* (Fig. 5C) and *C*. aff. *operosum* (Fig. 5F), but it was not clear whether this was a distinctive character.

## Discussion

### Ultrastructure

Ultrastructure studies of coccoid dinophyte cells are still rare because of many methodological problems [30], [38]. Nevertheless, they have great importance in better understanding the precise biological function of this specific developmental stage and gaining more basic data for phylogenetic reconstructions. Ecological stress such as temperature and light [43] and reduced availability of iron or nutrients may interfere with the interpretation of cellular ultrastructure [44]–[45]. We have aimed to avoid such bias by investigating cells held under constant culture conditions. Most of the cells studied here have shown subcellular details such as a reduced number of chloroplasts and an increased number of starch grains and lipid drops. These features have been interpreted as typical indicators for encysting cells or (early) coccoid cells, respectively [27], [30], [38], [46].

The number of layers constituting the shell of coccoid cells might have some phylogenetic significance [47]. *Thoracosphaera* [31]–[32] and *Leonella* Janofske & Karwath have a single matrix surrounding the cell (delineated by two unit membranes), while all extant species of *Scrippsiella* (including *S. minima* [30]) and its putative relatives such as *Calciodinellum* and *Pernambugia* share two layers (delineated by three unit membranes). These two types of shell architecture may correlate with molecular phylogenies, in which *Scrippsiella s.l*. and the T/Pf-clade represent two distinct lineages of the Thoracosphaeraceae [19]–[20]. As most peridinioid dinophytes exhibit the two-layer type [48]–[49], it is most plausible to assume that the one-layer type is apomorphic and today restricted to the T/Pf-clade. For future research, it is tempting to investigate the number of layers in coccoid cells of other members of the T/Pf-clade such as *Pfiesteria*.

For *S. minima*, Gao and co-workers [30] concentrated on mucofibrous material between the inner and the middle unit membranes surrounding the coccoid cell. This material is raised to form protrusions that are calcified in later stages of development. We have found structures similar in appearance in, for example, *Calciodinellum* and *Pernambugia*. They have also been documented (but neither described nor discussed) for *Thoracosphaera* [31] directly under the single matrix constituting the shell. Based on the relative position of such protrusions it is plausible to assume that the outer matrix in *Scrippsiella s.l*. is homologous to the single layer in the T/Pf-clade. To the best of our knowledge, protrusions of mucofibrous material as prerequisite for calcification [30] have not been reported outside the Thoracosphaeraceae, and it is therefore likely that they are apomorphic and play an important role during the biomineralization process.

In *Thoracosphaera*, calcification starts with the deposition of seed crystals in the single matrix present [31]–[32], while it is the outer matrix of *S. minima*, in which mineralization takes place [30]. This underlines once more the probable homology of both structures and is in accordance with the results presented here, as we never have observed crystal-like structures in an inner matrix. However, there are many fossil calcareous dinophytes known, particularly from the Mesozoic, with two distinct calcified layers that can be structurally differentiated [16], [50]. A direct comparison to the extant species is impossible, since all these forms have become extinct.

The presence of vacuoles including crystal-like particles has been previously reported from *Scrippsiella sweeneyae* Balech ex A.R.Loebl. [51] and *Th. heimii* [31], and the latter authors have assumed a crucial role of those structures during biomineralization. A striking observation of the present study is that vacuolar crystal-like particles are abundant among calcareous dinophytes (records for seven more species). They have also been documented in *Tyrannodinium edax* (A.J.Schill.) Calado ( = *Peridinium berolinense* Lemmerm. [35], a non-calcareous member of the T/Pf-clade [52]). However, vacuolar crystal-like particles have been sporadically reported (albeit under different names) from dinophytes (and also in thecate cells), but never have they been the focus of ultrastructural studies in a comparative approach. They have been found in the Gonyaulacales [53], Peridiniales [33]–[34], and Suessiales [24], [36], [38], [54], but their variation in size, shape, and subcellular distribution makes an overall homology unlikely.

### Chemistry and Function

Analytical chemistry of the crystal-like particles might likewise be indicative of their independent evolutionary origin, although the precise molecular composition based on ultimate analyses is rarely investigated. In the Suessiales, the vacuolar crystals with a characteristic rectangular shape are composed of calcium oxalate [55], while the bi-rhombohedral particles found in the Gonyaulacales contain guanine and other as yet unidentified components [56]. Conversely, the mature shell of calcareous dinophytes is composed of calcite elements, as it has been determined for *S. trochoidea* [12] and *Th. heimii* [57]. Inouye and Pienaar [31] have shown that the vacuolar crystal-like particles are sensitive to acid, and there is no reason to assume that they are not calcitic. For future research, the precise molecular composition of such particles found outside the Thoracosphaeraceae (in, e.g., *Galeidinium* Tam. & T.Horig. [34] and *Peridiniopsis* Lemmerm. [33]) is essential to reliably determine whether they are homologous across the Peridiniales.

Multiple functions of vacuolar crystal-like particles have been discussed. In the Suessiales, the mature elements are characteristically brick-like [24], [36], [58]–[59] and are associated with an eyespot in a regular arrangement of one to several rows [60]–[61]. Eyespots effectively absorb and reflect blue-green laser light [62] and are structurally connected to the flagellar apparatus. The support of these structures in locomotion has been therefore suggested [63]. More generally, the vacuoles containing crystal-like particles have been variously interpreted to be involved in the detoxification of the dinophyte cell [38], [53], [64]. Many calcifying organisms have access to corresponding physiological pathways to compose their aragonite and calcite structures [65]–[66]. Calcareous dinophytes may have thus modified the potential for detoxification to create calcitic shells for protection and/or as weight for sinking [67]. This specific function is particularly evident in such cavate coccoid cells of, for example, *Calcicarpinum* Deflandre, 1949 and *Posoniella* Streng, Banasová, Reháková & H.Willems. Such calcareous dinophytes are primarily found in surface sediments at coastal sites as the establishment of a dormancy seedbank [68]–[69].

### Conclusion

Compared to calcareous dinophytes, biomineralization in other unicellular organisms, such as the foraminifers and coccolithophores, has been more thoroughly investigated [9]–[10]. Foraminifers show principle differences in this process, as needle-like seed crystals are formed in vacuoles prior to the calcification of the shell (miliolid species), or not (hyaline species) [8]. At the ultrastructure level, the miliolid type somewhat resembles what is demonstrated for (calcareous) dinophytes in this and other studies. In coccolithophores, the crystallization process leading to the mature coccoliths takes place in Golgi-derived vesicles [10] moving from the cell center to the periphery.

The assumption that calcareous dinophytes have a similar calcification mechanism as coccolithophores has been postulated by Tangen and colleagues [32]. It is generally accepted that biomineralization in calcareous dinophytes also takes place under strong control at the cellular level [13], [70]–[71]. Tabulation patterns that are reflected in the shell of the coccoid cells in at least some members of the Thoracosphaeraceae indicate that biomineralization is linked to amphiesmal vesicles constituting the thecal plates. In coccoid cells at an early developmental stage, calcitic seed crystals are formed in vesicles that probably derive from the Golgi apparatus. Such vesicles are transported to the cell periphery, and the seed crystals are deposited in the outer (or only) matrix surrounding the coccoid cell. They may accumulate at collection sites, where they are visible as protrusions of mucofibrous material (functioning as ‘skeletons’: [30]). However, it remains unclear at present how the seed crystals pass through the inner matrix in *Scrippsiella* and its relatives. More research on the life history, ultrastructure, and physiology is necessary, and high-spatial resolution analyses such as NanoSIMS, Raman spectroscopy, soft X-ray microscopy, and atomic force microscopy may be promising approaches in developing a comprehensive scenario for biomineralization in calcareous dinophytes.
